# A Review of Computer-Aided Heart Sound Detection Techniques

**DOI:** 10.1155/2020/5846191

**Published:** 2020-01-10

**Authors:** Suyi Li, Feng Li, Shijie Tang, Wenji Xiong

**Affiliations:** ^1^College of Instrumentation and Electrical Engineering, Jilin University, Changchun, China; ^2^The First Hospital of Jilin University, Changchun, China

## Abstract

Cardiovascular diseases have become one of the most prevalent threats to human health throughout the world. As a noninvasive assistant diagnostic tool, the heart sound detection techniques play an important role in the prediction of cardiovascular diseases. In this paper, the latest development of the computer-aided heart sound detection techniques over the last five years has been reviewed. There are mainly the following aspects: the theories of heart sounds and the relationship between heart sounds and cardiovascular diseases; the key technologies used in the processing and analysis of heart sound signals, including denoising, segmentation, feature extraction and classification; with emphasis, the applications of deep learning algorithm in heart sound processing. In the end, some areas for future research in computer-aided heart sound detection techniques are explored, hoping to provide reference to the prediction of cardiovascular diseases.

## 1. Introduction

With the prevalence of unhealthy living habits, cardiovascular disease (CVD) has become one of the major threats to human health. According to the latest statistics of the World Health Organization (WHO) [1], the number of deaths from CVDs reached 17.9 million in 2016; CVD is the leading cause of mortality throughout the world. At present, there are about 290 million people suffering from cardiovascular diseases in China alone, so the prevention and treatment of cardiovascular diseases have become an urgent issue for health-conscious people.

Heart sounds—the sounds made by the heart systole and diastole—can be recorded as heart sound signals, also known as phonocardiography (PCG), whose acquisition is noninvasive and easy. Through PCG data processing and analyzing, the results can be used as an assistant diagnostic tool for the prediction of cardiovascular diseases. However, due to the characteristics of the heart sound signals and the influence of the noise in the environment, the detection of heart sound signals is facing great challenges. On the one hand, the randomness and variability of cardiovascular disease symptoms lead to the complexity and diversity in the signal manifestation. On the other hand, heart sound signals are relatively weak, and the acquisition process of the original signals can be affected by various noises and interferences, resulting in noisy data collected, which can reduce the accuracy of related parameter extractions and increase the uncertainty of diagnosis.

Computer-aided detection technology is a fast, efficient and economical tool [2], which can be applied to quantitative acquisition and the analysis of heart sound signals. By extracting the key parameters in the PCG and comparing the patient's monitoring sequence with the tagged database, not only can more intuitive diagnostic results be obtained automatically, but the potential cardiovascular disease may be further inferred by the experts with their clinical knowledge. In recent years, computer-aided detection technology for the heart sound signals processing and analysis has made remarkable achievements and aroused wide interest [3, 4].

At present, intelligent auscultation technology has not been widely used in clinical diagnosis, and the main method used for heart sound detection is manual auscultation. Therefore, the research and application of computer-aided techniques for heart sound detection will greatly promote development in the field of cardiovascular disease diagnosis.

The purpose of this paper is to provide an overview of computer-aided heart sound detection techniques in recent years. The clinical characteristics of heart sound signals are introduced, first. Then, some promising processing and analyzing techniques for heart sound detection that have developed over the last five years are reviewed. Next, the deep learning algorithm that can be applied to the PCG processing and analysis is discussed. Finally, some promising research areas in computer-aided heart sound detection techniques are recommended.

## 2. Heart Sounds and Cardiovascular Diseases

**Figure 1:**
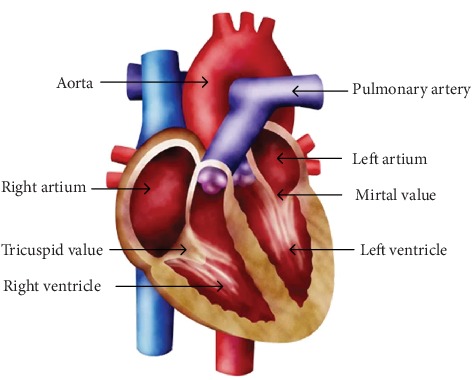
Section view of the heart. The heart valves and arteries associated with auscultation are marked.

**Figure 2:**
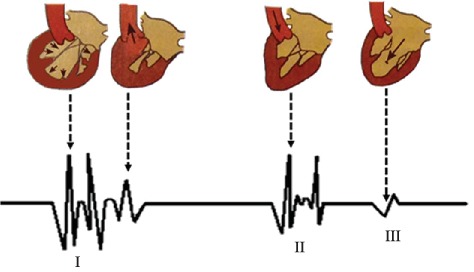
Graphical representation of partial heart sound components and the corresponding changes in the direction of blood flow in the heart.

Vibrations caused by cardiac activities such as myocardial contraction, heart valve closure, and occlusion of the ventricular wall are transmitted through the tissue to the surface of the chest wall and form the heart sound signals that can be perceived by the human ear and recorded with electronic instruments.  Figure 1 shows the location of heart valves and arteries associated with auscultation. According to the order of occurrence in a cardiac cycle, the heart sound is divided into four components: the first heart sound (S1), the second heart sound (S2), the third heart sound (S3) and the fourth heart sound (S4). For each of the 4 components, the physiological state of the heart is different.  Figure 2 shows the blood flow changes of partial heart sound components in the heart. The intensity, frequency and correlation of the heart sound reflect the heart valve condition, myocardial function and intracardiac blood flow.  Table 1 shows the mechanism of the generation of heart sounds, including the cause, features and significance of heart sounds [5].

**Figure 3:**
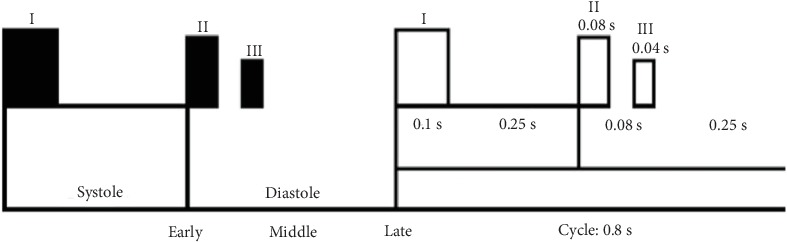
Heart sounds and cardiac cycles. The duration of S1, S2 and S3 and the relationship between systole and diastole in the heart are marked.

The fundamental heart sounds (FHS) [6] used in clinical diagnosis include S1 and S2 (S3 appears only in the cardiac cycles of some healthy young people, and S4 does not appear in normal cardiac cycles). The period between S1 and S2 in the same cardiac cycle is called systole, and the one between S2 and S1 in the next cycle is called diastole. The normal duration of systole is about 0.35 sec and that of diastole is about 0.45 sec, for a total of 0.8 sec in a complete cycle. These values are closely related to the occurrence of cardiovascular diseases.  Figure 3 shows two normal cardiac cycles.

Heart sound diagnosis with manual auscultation is a qualitative method entirely based on the experience of the expert through analysis of the tone and intensity of the heart sounds. Computer-aided detection techniques for heart sound analysis can obtain the quantized characteristic parameters, which are helpful to find the relationship between the heart sounds and the related diseases. It is conducive to the subsequent traceability of data and the formation of database as well. Therefore, it is significant to research in the non-invasive diagnosis of cardiovascular disease.

## 3. Computer-Aided Heart Sound Detection Techniques

The computer-aided processing of heart sounds includes denoising [7], segmentation [8], feature extraction and classification [9].

### 3.1. Denoising

Due to the influence of the external environment, heart sound signals are usually coupled with electromagnetic interference, power frequency interference, random noise, interference from the human body, breath sounds, and lung sounds [10]. The diagnostic accuracy of the detection is directly affected by the quality of the signals and the features extracted subsequently. Consequently, denoising is the first essential step to improve the automatic detection accuracy of heart sounds. The techniques used for heart sound denoising include discrete wavelet transform (DWT), adaptive filtering denoising, singular value decomposition (SVD), etc. In addition, combined methods are applied for better effects, which help to improve the signal quality and detection accuracy.

Jain et al. [11] proposed a DWT-based PCG signal denoising algorithm, using “Coif-5” wavelet as the mother wavelet and combined with an adaptive threshold estimation method, a nonlinear intermediate function method and a genetic algorithm, to optimize the traditional discrete wavelet transform (DWT) algorithm. The improved algorithm eliminated the out-of-band noises and removed the lower detail level coefficients, further improving the denoising performance. Mondal et al. [12] introduced a novel heart-tone denoising method based on the combined framework of wavelet packet transformation and SVD. According to the standard of mutual information measurement, the most abundant nodes in the wavelet tree were selected, and the noise component from the heart sound signals was suppressed by using the SVD technique to process the coefficients corresponding to the selected nodes. Ali et al. [13] selected different DWT families, threshold types, and signal decomposition levels to denoise the heart sound signals, and evaluated the influence of different wavelet functions and wavelet decomposition levels on the efficiency of the denoising algorithm. They concluded that the Db10 wavelet and the discrete Meyer wavelet with the fourth-order decomposition can obtain the maximum SNR (signal-to-noise ratio) and the minimum RMSE (standard error) of the standard heart sounds. Zheng et al. [14] proposed an innovative denoising framework based on a combination of modified SVD and Compressed Sensing (CS), which can well maintain the original morphological characteristics of heart sounds. Compared with the traditional techniques such as DWT and empirical mode decomposition (EMD), this framework can obtain a larger SNR. The denoised heart sound signals still had the highest correlation with the original heart sound signals. Deng and Han [15] proposed an adaptive denoising algorithm. Compared with the conventional wavelet method, the proposed algorithm had better denoising effect.

### 3.2. Segmentation

Segmentation is often performed on the raw signal or the denoised signal. The purpose of segmentation is to find the beginning and end of heart sounds, and to segment S1, S2, systole, and diastole for the subsequent feature extraction. To date, the methods used for heart sounds segmentation mainly include hidden Markov models (HMM), WT, and correlation coefficient matrices, etc.  Table 2 summarizes some of the heart sound segmentation literature in the past five years.

### 3.3. Feature Extraction and Classification

The goal of feature extraction is to find out a small number of representative features to replace the high-dimensional raw signals. In general, the classification model based on features training is more efficient and accurate than that which is based on raw signals training. Feature extraction is often performed on the signal with segmentation. DWT, continuous wavelet transformation (CWT), short-time Fourier transform (STFT) and Mel Frequency Cepstrum Coefficient (MFCC) are commonly used methods for heart sounds feature extraction. Without segmentation, feature extraction can be conducted on the raw signal or the denoised signal.

Classification can be performed on the features, the raw signals and the denoised signals as well. The goal of classification is to present the qualitative results of the detection, dividing the heart sound signals into the normal or abnormal. The classification techniques for heart sounds include HMM, Support Vector Machine (SVM), Artificial Neural Networks (ANN), k-Nearest Neighbor (kNN), Euclidean distance, etc.  Table 3 lists the representative literature on the feature extraction and classification of the heart sound signals over the past five years.

These techniques (SVM, kNN, BP neural network, and logistic regression) all utilize machine learning—an algorithm that allows computer systems to effectively access and analyze data to adjust and improve functioning based on patterns and experience, without the need for explicit programming. In recent years, machine learning has been widely used in heart sound classification. As the incidence of cardiovascular disease increases, the amount of heart sound data to be processed is also increasing. In order to ensure the accuracy of classification while processing a large amount of data, deep learning algorithm has emerged.

## 4. Application of Deep Learning in Heart Sound Classification

Deep learning is a branch of machine learning that imitates the workings of the human brain, through artificial neural networks—complex algorithms inspired by the brain itself. Thus, it can automatically extract the characteristics of original signals and find out the rules among data by means of a deeper learning than the traditional machine learning, thereby improving its accuracy and efficiency of classification. The concept of deep learning was proposed by Hinton et al. [49] in 2006. Deep learning utilizes the relative relationship of space, and combines low-level models to form more complex high-level models, which greatly improves the training performance of the system. In recent years, it has shown good practicality and reliability in the fields of speech recognition [50], image recognition [51], biomedical data analysis [52, 53], signal processing [54], automatic driving [55] and other areas. Deep learning models have been applied to classify heart sound signals, and the models mainly include Deep Neural Networks (DNN), Convolution Neural Networks (CNN), Recurrent neural networks (RNN) and etc.  Table 4 lists the representative literature on the deep learning applied in the classification of heart sound signals over the past five years.

Deep learning has shown good superiority in the computer-aided classification of heart sound signals, but it also faces some challenges. First of all, there are too many parameters of the deep learning model, with a large amount of data to be optimized, a long execution time and a large training data set required. Secondly, the deep learning modelling calls for higher configuration of the computer with powerful CPU and GPU for calculation, hence the experiment cost is high, and the model is unsuitable for home computers and microcomputers. However, the portable heart sound devices have great development potential and good application prospects.

## 5. Conclusions

With the increasing incidence of cardiovascular diseases in recent years, a greater attention has been drawn to non-invasive heart sound detection technology. In this study, the latest research on computer-aided heart sound detection techniques over the last five years has been reviewed, with the applications of deep learning to the heart sound classification as an emphasis.

Regarding the potential contributions of the technology to human health promotion, the following areas for future research are recommended. A large amount of heart sound data is needed to supplement the heart sound database. Heart sound data is a reliable source of information for discovering the hidden features of the cardiovascular diseases. Therefore, it is necessary to complete and improve the heart sound database and its corresponding expert annotations, for better model training and a more accurate assistant diagnose. Since large-scaled computer systems are already available in hospitals, it has become feasible to establish the complex deep learning model, which will be able to process the heart sound data. Thus, the data processing and the parameters optimizing techniques need more in-depth study. The deep learning modeling requires higher computer configurations with powerful GPU support, but the compressed deep learning algorithms can work on PC or microcomputers. Since the heart sounds classification model based on compressed deep learning algorithms are more accurate than those based on traditional algorithms, further study on the heart sound classification model based on the compressed deep learning algorithms is helpful to the popularization and application of portable heart sound detection.

## Figures and Tables

**Table 1:** The characteristics and significance of heart sounds.

Heart sound	Cause	Features	Significance
S1	Closure of the mitral (M1) and tricuspid (T1) valves, opening of the semilunar valve.	Frequency: 50–150 Hz	For the diagnosis of ventricular contractility and atrioventricular valve function, myocarditis, cardiomyopathy, myocardial infarction or heart failure disease.
		Time: 50–100 ms	
S2	Deceleration of blood flow in the aorta and pulmonary artery, closure of the semilunar valve, opening of the atrioventricular valve.	Frequency: 50–200 Hz	Relates to the functional state of arterial wall, high/low blood pressure, atherosclerosis, pulmonary heart disease, primary/pulmonary stenosis, left-to-right shunt congenital heart disease.
		Time: 25–50 ms	
S3	The blood flowing rapidly from the ventricle impacts the wall of the chamber from the atrium, causing sudden tension and vibration of the ventricular wall, chordae and papillary muscles.	Frequency: 25–70 Hz	Appears in some healthy young people.
		Time: 120–150 ms	
S4	Tension and vibration caused by atrioventricular valve and its related structures.	Frequency: <30 Hz	Belongs to pathological heart sounds, appears in some elderly populations and people with early coronary heart disease.
		Time: before S1 about 90 ms	

**Table 2:** Segmentation methods of PCG signals.

Year	Author	Segmentation method	Dataset	Result		
2019	Giordano and Knaflitz [16]	Envelope-based technique	Sample population of 24 healthy subjects over 10-min-long simultaneous phonocardiography recordings	F1 of 99.2%		
2019	Oliveira et al. [17]	HSMM-GMM	PhysioNet [18], PASCAL [19] and a pediatric dataset composed of 29 heart sounds	*F-*score of 92%		
2019	Kamson et al. [20]	HSMM	Training-set-a of 2016 PhysioNet/computing in cardiology challenge	Sensitivity	*P*+	F1
				98.28	98.45	98.36
2019	Renna et al. [21]	HSMM-CNN	PhysioNet	Sensitivity: 93.9%		
2018	Liu et al. [22]	Time-domain analysis, frequency-domain analysis and time-frequency-domain analysis	Heart sound & Murmur library of UMich	Sensitivity: 98.63%		
2018	Belmecheri et al. [23]	Correlation coefficients matrix	A database of 21 clean heart sounds	Sensitivity: 76%		
2018	Alexander et al. [24]	HMM	3240 PCG recordings from PhysioNet and PASCAL	Sensitivity	Specificity	
				90.3%	89.9%	
2017	Babu et al. [25]	VMD	Database:	Sensitivity	*P*+	Accuracy
			PhysioNet	98.90	96.07	95.14
			PASCAL	99	100	99
			Michigan [26]	100	100	100
			eGeneralMedical [27]	100	100	100
			Real-time PCG signals	100	97.08	97.08
2017	Varghees et al. [28]	EWT	PhysioNet, PASCAL, Michigan, eGeneralMedical and real-time PCG signals	Sensitivity	Pp	OA
				94.38%	97.25%	91.92%
2017	Liu et al. [29]	HSMM	More than 120 000 s of heart sounds recorded from eight independent heart sound databases	F1 of 98.5%		
2016	Thomas et al. [30]	Fractal decomposition (FD)	Michigan (23 different heart sounds and 6 patients' recordings done in a real clinical environment)	Sensitivity	+*P*	DER
				96.97	99.58	3.55
2016	Springer et al. [31]	HSMM	405 synchronous 30–40 s PCG and ECG recordings from 123 deidentified adult patients	F1 of 95.63 ± 0.85%		
2015	Salman et al. [32]	Peak intervals pattern	1089 cycles from 62 set of normal and abnormal signals	Correct cycle detected rate of 83.38%		

**Table 3:** Feature extraction and classification methods of PCG signals.

Year	Author	Feature extraction methods	Classifier	Database	Result		
2019	Shi et al. [33]	Feature extraction algorithm of Springer	AdaBoost	PhysioNet and PASCAL	ACC: 96.36%		
2019	Nogueira et al. [34]	MFCC	SVM	PhysioNet	Sensitivity	Specificity	Accuracy
					91.87%	82.05%	97%
2019	Cheng (without segmentation) [35]	Envelope autocorrelation	SVM	HSCT11 dataset	Accuracy all could reach to 100%		
2018	Meintjes et al. [36]	CWT	SVM, kNN	PhysioNet	MAcc: 86%		
2018	Hamidi et al. [37]	Curve fitting, MFCC	Euclidean distance	Dataset A from PhysioNet	MAcc: 92%		
				Dataset B from PhysioNet	MAcc: 81%		
				Dataset C from PhysioNet	MAcc: 98%		
2018	Juniati et al. [38]	DWT	kNN, Fuzzy c-means clustering	40 normal heart sounds, 40 extra systole, 40 murmurs	MAcc: 86.17%		
2017	Kay et al. [39]	CWT, MFCC	BP neural networks	PhysioNet	MAcc: 85.2%		
2017	Karar et al. [40]	DWT	Rule-based classification tree	22 sets of heart sounds and noise data from the public database of the CliniSurf medical school	MAcc: 95.5%		
2017	Zhang et al. [41]	Tensor decomposition	SVM	Dataset A: normal heart sounds, extra systole, murmurs, artificial heart sounds	MAcc: 76%		
				Dataset B: normal heart sounds, extra systole, murmurs	MAcc: 83%		
				Dataset C: normal heart sounds, abnormal heart sounds	MAcc: 88%		
2017	Langley and Murray (without segmentation) [42]	/	Wavelet entropy	PhysioNet	Sensitivity	Specificity	Accuracy
					94%	65%	80%
2017	Whitaker et al. [43]	Sparse coding	SVM	PhysioNet	Sensitivity	Specificity	MAcc
					84.3%	77.2%	80.7%
2017	Li et al. [44]	FFT	BP neural networks	PhysioNet	Sensitivity	Specificity	MAcc
					68.36%	94.01%	88.56%
			Logistic regression		Sensitivity	Specificity	MAcc
					75.68%	87.71%	72.56%
2016	Deng and Han (without segmentation) [45]	DWT	SVM-DM	Dataset A from PASCAL	The highest total precision of 3.17		
				Dataset B from PASCAL	The highest total precision of 2.03		
2015	Zheng et al. [46]	EMD	SVM	A dataset collected from the healthy volunteers and CHF patients	Sensitivity	Specificity	Accuracy
					96.59%	93.75%	95.39%
2015	Safara [47]	Wavelet packet tree	Higher-order cumulants (HOC)	A set of 59 heart sounds from different categories: normal heart sounds, mitral regurgitation, aortic stenosis, and aortic regurgitation.	Best classification accuracies: 99.39%		
2011	Yuenyong et al. (without segmentation) [48]	DWT	Neural network	Several on-line databases and recorded with an electronic stethoscope	Tenfold cross-validation: 0.92 for noise free case, 0.90 under white noise with 10 dB signal-to-noise ratio (SNR), and 0.90 under impulse noise up to 0.3 s duration		

**Table 4:** Literature for heart sound classification using deep learning.

Year	Author	Segmentation method	Dataset	Performance				
2019	Wu et al. [56]	CNN	PhysioNet (2575 normal heart sounds and 665 abnormal heart sounds)	Hold out testing				
				Sensitivity	Specificity		Accuracy	
				86.46%	85.63%		86.0%	
				Ten-fold cross validation				
				Sensitivity	Specificity		Accuracy	
				91.73%	87.91%		89.81%	
2019	Abduh et al. [57]	DNN	PhysioNet	Sensitivity	Specificity		Accuracy	
				89.30%	97%		95.50%	
2018	Gharehbaghi and Lindén [58]	DTGNN	130 recordings of the heart sound signal	Sensitivity	Specificity		CR	
				83.9%	86%		85.5%	
2018	Chen et al. [59]	DNN	PASCAL	Sensitivity	Specificity		Accuracy	Precision
				98%	88.5%		93%	89.1%
2018	Yaseen et al. [60]	DNN	5 categories of heart sound signal, 200 per class (N, AS, MR, MS, MVP)	Sensitivity	Specificity			
				94.5%	98.2%			
2018	Han et al. [61]	CNN	2575 normal recordings and 665 abnormal recordings	MAcc	Sensitivity		Specificity	
				91.50%	98.33%		84.67%	
2018	Ren et al. [62]	CNN	PhysioNet	19.8% higher than the baseline accuracy obtained using traditional audio processing functions and support vector machines.				
2018	Morales et al. [63]	CNN	PhysioNet	Accuracy	Sensitivity		Specificity	
				97%	93.20%		95.12%	
2018	Baris et al. [64]	CNN	UoC-murmur database (innocent murmur versus pathological Murmur) and PhysioNet-2016 database (normal versus pathological)	MAcc	Specificity		Sensitivity	
				81.5%	78.5%		84.5%	
2018	Messner et al. [65]	DNN	PhysioNet	F1 ≈ 96%				
2017	Ghaemmaghami et al. [66]	DNN	128 recordings from male and female subjects with healthy hearts	Accuracy	Sensitivity		Specificity	
				95.8%	83.2%		99.2%	
2017	Sujadevi et al. [67]	RNN & LSTM&GRU	Dataset A from PhysioNet			Accuracy	Precision	
				RNN 4 layer		53.8%	55.8%	
				LSTM 4 layer		76.9%	83.3%	
				GRU 4 layer		75.3%	78.2%	
			Dataset B from PhysioNet			Accuracy	Precision	
				RNN 4 layer		65.2%	68.1%	
				LSTM 4 layer		74.7%	94.5%	
				GRU 4 layer		74.4%	69.7%	
2017	Chen et al. [68]	DNN	311 S1 and 313 S2 from 16 people (11 males and 5 females)	Accuracy: 91.12%				
2017	Yang and Hsieh [69]	RNN	PhysioNet	MAcc: 84%				
2017	Zhang and Han [70]	CNN	Dataset A from PASCAL	Normalized precision: 0.77				
			Dataset B from PASCAL	Normalized precision: 0.71				
2017	Faturrahman et al. [71]	DBN	MITHSDB [72]	Accuracy: 84.89%				
			AADHSDB [73]	Accuracy: 86.15%				
2017	Maknickas and Maknickas [74]	CNN	PhysioNet	Train accuracy: 99.7%				
				Validation accuracy: 95.2%				
2016	Thomae et al. [75]	DNN	PhysioNet	Sensitivity	Specificity		Score	
				96%	83%		0.89	
2016	Tschannen and Dominik [76]	CNN	PhysioNet	Sensitivity	Specificity		Score	
				84.8%	77.6%		0.812	
2016	Potes et al. [77]	AdaBoost & CNN	PhysioNet	Sensitivity	Specificity		MAcc	
				94.24%	77.81%		86.02%	

## References

[1] Geneva: World Health Organization (2018). *World Health Statistics 2018: Monitoring Health for the SDGs, Sustainable Development Goals*.

[2] Mahnke C. B. Automated heartsound analysis, computer-aided auscultation: a cardiologist’s perspective and suggestions for future development.

[3] Wang J., Lo C., Wu C. Computer-aided analysis and classification of heart sounds based on neural networks and time analysis.

[4] Watrous R. L. Computer-aided auscultation of the heart: from anatomy and physiology to diagnostic decision support.

[5] Cheng X., Zhang Z. (2014). Denoising method of heart sound signals based on self-construct heart sound wavelet. *AIP Advances*.

[6] Abdollahpur M., Ghaffari A., Ghiasi S., Mollakazemi M. J. (2017). Detection of pathological heart sounds. *Physiological Measurement*.

[7] Salman A. H., Ahmadi N., Mengko R., Langi A. Z. R., Mengko T. L. R. Performance comparison of denoising methods for heart sound signal.

[8] Suboh M. Z., Yaakop M., Yid M. S. M., Morad M. H. C. Segmentation of heart sound signals into cycles based on peak intervals pattern.

[9] Chakir F., Jilbab A., Nacir C., Hammouch A. (2018). Phonocardiogram signals processing approach for PASCAL classifying heart sounds challenge. *Signal, Image and Video Processing*.

[10] Salman A. H., Ahmadi N., Mengko R., Langi A. Z. R., Mengko T. L. R. Performance comparison of denoising methods for heart sound signal.

[11] Jain P. K., Tiwari A. K. An adaptive method for shrinking of wavelet coefficients for phonocardiogram denoising.

[12] Mondal A., Saxena I., Tang H., Banerjee P. (2018). A noise reduction technique based on nonlinear kernel function for heart sound analysis. *IEEE Journal of Biomedical and Health Informatics*.

[13] Ali M. N., El-Dahshan E. S. A., Yahia A. H. (2017). Denoising of heart sound signals using discrete wavelet transform. *Circuits Syst Signal Process*.

[14] Zheng Y., Guo X., Jiang H., Zhou B. (2017). An innovative multi-level singular value decomposition and compressed sensing based framework for noise removal from heart sounds. *Biomedical Signal Processing & Control*.

[15] Deng S. W., Han J. Q. (2018). Adaptive overlapping-group sparse denoising for heart sound signals. *Biomedical Signal Processing and Control*.

[16] Giordano N., Knaflitz M. (2019). A novel method for measuring the timing of heart sound components through digital phonocardiography. *Sensors*.

[17] Oliveira J., Renna F., Mantadelis T., Coimbra M. (2019). Adaptive sojourn time HSMM for heart sound segmentation. *IEEE Journal of Biomedical and Health Informatics*.

[18] PhysioNet. https://physionet.org/.

[19] Bentley P., Nordehn G., Coimbra M., Mannor S., Getz R. (2015). Classifying heart sounds challenge (PASCAL database). http://www.peterjbentley.com/heartchallenge/index.html.

[20] Kamson A. P., Sharma L. N., Dandapat S. (2019). Multi-centroid diastolic duration distribution based HSMM for heart sound segmentation. *Biomedical Signal Processing and Control*.

[21] Renna F., Oliveira J. H., Coimbra M. T. (2019). Deep convolutional neural networks for heart sound segmentation. *IEEE Journal of Biomedical and Health Informatics*.

[22] Liu Q. S., Wu X. M., Ma X. J. (2018). An automatic segmentation method for heart sounds. *Biomedical Engineering Online*.

[23] Belmecheri M. Z., Ahfir M., Kale I. (2018). Automatic heart sounds segmentation based on the correlation coefficients matrix for similar cardiac cycles identification. *Biomedical Signal Processing and Control*.

[24] Alexander B., Nallathambi G., Selvaraj N. Screening of heart sounds using hidden Markov and Gammatone filterbank models.

[25] Babu K. A., Ramkumar B., Manikandan M. S. S1 and S2 heart sound segmentation using variational mode decomposition.

[26] Judge R. D., Mangrulkar R. (2015). *The Open Michigan Heart Sound & Murmur Library (OMHSML)*.

[27] Cardiac Auscultation of Heart Murmurs database (2017). http://www.egeneralmedical.com/listohearmur.html.

[28] Varghees V. N., Ramachandran K. I. (2017). Effective heart sound segmentation and murmur classification using empirical wavelet transform and instantaneous phase for electronic stethoscope. *IEEE Sensors Journal*.

[29] Liu C., Springer D., Clifford G. D. (2017). Performance of an open-source heart sound segmentation algorithm on eight independent databases. *Physiological Measurement*.

[30] Thomas R., Hsi L. L., Boon S. C., Gunawan E. Heart sound segmentation using fractal decomposition.

[31] Springer D. B., Tarassenko L., Clifford G. D. (2016). Logistic Regression-HSMM-Based Heart Sound Segmentation. *IEEE Transactions on Biomedical Engineering*.

[32] Salman A. H., Ahmadi N., Mengko R., Langi A. Z. R., Mengko T. L. R. Automatic segmentation and detection of heart sound components S1, S2, S3 and S4.

[33] Shi K., Schellenberger S., Michler F. (2019). Automatic signal quality index determination of radar-recorded heart sound signals using ensemble classification. *IEEE Transactions on Biomedical Engineering*.

[34] Nogueira D. M., Ferreira C. A., Gomes E. F., Jorge A. M. (2019). Classifying heart sounds using images of motifs, MFCC and temporal features. *Journal of Medical Systems*.

[35] Cheng X., Zhan Q., Wang J., Ma R. A high recognition rate of feature extraction algorithm without segmentation.

[36] Meintjes A., Lowe A., Legget M. Fundamental heart sound classification using the continuous wavelet transform and convolutional neural networks.

[37] Hamidi M., Ghassemian H., Imani M. (2018). Classification of heart sound signal using curve fitting and fractal dimension. *Biomedical Signal Processing and Control*.

[38] Juniati D., Khotimah C., Wardani D. E. K., Budayasa K. (2018). Fractal dimension to classify the heart sound recordings with KNN and fuzzy c-mean clustering methods. *Journal of Physics Conference Series*.

[39] Kay E., Agarwal A. (2017). DropConnected neural networks trained on time-frequency and inter-beat features for classifying heart sounds. *Physiological Measurement*.

[40] Karar M. E., El-Khafif S. H., El-Brawany M. A. (2017). Automated diagnosis of heart sounds using rule-based classification tree. *Journal of Medical Systems*.

[41] Zhang W., Han J., Deng S. (2017). Heart sound classification based on scaled spectrogram and tensor decomposition. *Expert Systems with Applications*.

[42] Langley P., Murray A. (2017). Heart sound classification from unsegmented phonocardiograms. *Physiological Measurement*.

[43] Whitaker B. M., Suresha P. B., Liu C., Clifford G. D., Anderson D. V. (2017). Combining sparse coding and time-domain features for heart sound classification. *Physiological Measurement*.

[44] Li L., Wang X., Du X., Liu Y., Liu C., Qin C. Classification of heart sound signals with BP neural network and logistic regression.

[45] Deng S. W., Han J. Q. (2016). Towards heart sound classification without segmentation via autocorrelation feature and diffusion maps. *Future Generation Computer Systems*.

[46] Zheng Y., Guo X., Qin J., Xiao S. (2015). Computer-assisted diagnosis for chronic heart failure by the analysis of their cardiac reserve and heart sound characteristics. *Computer Methods and Programs in Biomedicine*.

[47] Safara F. (2015). Cumulant-based trapezoidal basis selection for heart sound classification. *Medical & Biological Engineering & Computing*.

[48] Yuenyong S., Nishihara A., Kongprawechnon W., Tungpimolrut K. (2011). A framework for automatic heart sound analysis without segmentation. *Biomedical Engineering Online*.

[49] Hinton G., Osindero S., Welling M., Teh Y. (2006). Unsupervised discovery of nonlinear structure using contrastive backpropagation. *Cognitive Science*.

[50] Deng L., Li J., Huang J. T., Yao K., Yu D., Seide F. Recent advances in deep learning for speech research at Microsoft.

[51] Sun Y., Wang X., Tang X. Deep learning face representation from predicting 10,000 classes.

[52] Alipanahi B., Delong A., Weirauch M. T., Frey B. J. (2015). Predicting the sequence specificities of DNA- and RNA-binding proteins by deep learning. *Nature Biotechnology*.

[53] Shen D., Wu G., Suk H. I. (2017). Deep Learning in Medical Image Analysis. *Annual Review of Biomedical Engineering*.

[54] Yu D., Deng L. (2011). Deep learning and its applications to signal and information processing [exploratory DSP]. *IEEE Signal Processing Magazine*.

[55] Liu M., Niu J., Wang X. An autopilot system based on ROS distributed architecture and deep learning.

[56] Wu J. M. T., Tsai M. H., Huang Y. Z. (2019). Applying an ensemble convolutional neural network with Savitzky–Golay filter to construct a phonocardiogram prediction model. *Applied Soft Computing*.

[57] Abduh Z., Nehary E. A., Wahed M. A., Kadah Y. M. (2019). Classification of heart sounds using fractional fourier transform based mel-frequency spectral coefficients and stacked autoencoder deep neural network. *Journal of Medical Imaging and Health Informatics*.

[58] Gharehbaghi A., Lindén M. (2018). A deep machine learning method for classifying cyclic time series of biological signals using time-growing neural network. *IEEE Transactions on Neural Networks and Learning Systems*.

[59] Chen L., Ren J., Hao Y., Hu X. (2018). The diagnosis for the extrasystole heart sound signals based on the deep learning. *Journal of Medical Imaging and Health Informatics*.

[60] Yaseen, Son G. Y., Kwon S. (2018). Classification of heart sound signal using multiple features. *Applied Sciences-Basel*.

[61] Han W., Yang Z., Lu J., Xie S. (2018). Supervised threshold-based heart sound classification algorithm. *Physiological Measurement*.

[62] Ren Z., Cummins N., Pandit V., Han J., Qian K., Schuller B. Learning image-based representations for heart sound classification.

[63] Dominguez-Morales J. P., Jimenez-Fernandez A. F., Dominguez-Morales M. J., Jimenez-Moreno G. (2018). Deep neural networks for the recognition and classification of heart murmurs using neuromorphic auditory sensors. *IEEE Transactions on Biomedical Circuits and Systems*.

[64] Baris B., Ioannis G., Yannis S. (2018). A study of time-frequency features for CNN-based automatic heart sound classification for pathology detection. *Computers in Biology and Medicine*.

[65] Messner E., Zöhrer M., Pernkopf F. (2018). Heart sound segmentation—an event detection approach using deep recurrent neural networks. *IEEE Transactions on Biomedical Engineering*.

[66] Ghaemmaghami H., Hussain N., Tran K. Automatic segmentation and classification of cardiac cycles using deep learning and a wireless electronic stethoscope.

[67] Sujadevi V. G., Soman K. P., Vinayakumar R., Sankar A. U. P. Deep models for phonocardiography (PCG) classification.

[68] Chen T., Yang S., Ho L. (2017). S1 and S2 heart sound recognition using deep neural networks. *IEEE Transactions on Biomedical Engineering*.

[69] Yang T. C. I., Hsieh H. Classification of acoustic physiological signals based on deep learning neural networks with augmented features.

[70] Zhang W. J., Han J. Q. Towards heart sound classification without segmentation using convolutional neural network.

[71] Faturrahman M., Wasito I., Ghaisani F. D., Mufidah R. A classification method using deep belief network for phonocardiogram signal classification.

[72] Syed Z., Leeds D., Curtis D., Nesta F., Levine R. A., Guttag J. (2007). A framework for the analysis of acoustical cardiac signals. *IEEE Transactions on Biomedical Engineering*.

[73] Schmidt S. E., Holst-Hansen C., Hansen J., Toft E., Struijk J. J. (2015). Acoustic features for the identification of coronary artery disease. *IEEE Transactions on Biomedical Engineering*.

[74] Maknickas V., Maknickas A. (2017). Recognition of normal–abnormal phonocardiographic signals using deep convolutional neural networks and mel-frequency spectral coefficients. *Physiological Measurement*.

[75] Thomae C., Dominik A. Using deep gated RNN with a convolutional front end for end-to-end classification of heart sound.

[76] Tschannen M., Kramer T., Marti G., Heinzmann M., Wiatowski T. Heart sound classification using deep structured features.

[77] Potes C., Parvaneh S., Rahman A., Conroy B., America P. N., Solutions A. C. Ensemble of feature-based and deep learning-based classifiers for detection of abnormal heart sounds.

